# Pain and apathy

**DOI:** 10.1590/S1980-57642009DN20400023

**Published:** 2008

**Authors:** Valéria Santoro Bahia

**Affiliations:** MD, PhD. Behavioral and Cognitive Neurology Unit, Department of Neurology, Hospital das Clínicas, University of São Paulo School of Medicine, São Paulo, Brazil.

**Keywords:** pain, apathy, frontotemporal, dementia, anterior cingulate cortex

## Abstract

In this case report we discuss the lack of emotional reactivity and evasive motor motivation to nociceptive stimuli presented by a patient with frontotemporal degenerative disease and apathy as a predominant behavioral symptom.

## Abstract (pt)

Neste relato de caso, nós discutimos a falta de reatividade emocional e motora frente a um forte estímulo doloroso apresentada por uma paciente com doença degenerativa fronto-temporal cujo principal sintoma era a apatia.

Pain is an unpleasant sensory and emotional experience associated with real or potential tissue damage, and can be modulated by cognitive factors such as previous experience and attention.^1^

There are several scales involving self-report, informant rating and direct observations for assessment of pain in patients with dementia.^2-5^ Patients with dementia in early stages are able to complete at least one of the available pain scales but the reliability of these scales decreases with progressive decline in cognitive function which leads to underdetection and undertreatment of pain despite a very high prevalence of pain problems in this population.^6,7^

This situation also causes increased levels of stress in caregivers as the pain may trigger behavioral disturbances especially in patients with severe dementia^8^. In view of this, systematic pain assessment in this patient group is considered important.^9^

In patients with dementia, it remains unclear if the difficulty in the management of pain results from impaired communication and memory of pain, or whether the perception and experience of pain is altered as a result of the progressive degeneration of cortical and subcortical regions involved in the transmission and processing of nociceptive information.^10,11^

## Case report

A 52 year-old Portuguese woman, with three years of schooling, presented with a 3-year history of behavioral changes and cognitive impairment. Relatives reported that the disease started with stereotypical movements in the right hand and leg followed by aggressiveness. It was noted that she would keep talking about the same issue, and over time began to speak less and less, developing changes in the pitch of her voice. She was not interested in house chores or family problems and started to overeat.

The patient was medicated by a psychiatrist with haloperidol and risperidone due to clinical suspicion of Huntington’s disease or psychosis. The medication improved the aggressiveness, but not the stereotyped movements. The deterioration of the clinical picture was progressive and relatively fast.

Her father had died with dementia and behavioral changes, but a precise diagnosis was never documented. Her brother had also received assistance at our outpatient clinic for a diagnosed frontotemporal dementia.

During the clinical examination at her first evaluation at the Behavioral and Cognitive Neurology Unit, Department of Neurology, HC-FMUSP, she was apathetic. She spoke only after great insistence and was hypophonic. She displayed stereotyped movements slapping her hands on her knees, bringing her legs together, and stood up from the chair several times.

She kept her upper limb flexed held to her chest, showing mild hypertonia. Her gait was unstable with a tendency to fall in any direction. No alterations in ocular movement were evident.

She was dependent for all activities (Functional Activity Questionnaire^12^ scoring 22 out of a maximum 30; controls score 0–2). She could not take food to her mouth and had to be spoon fed.

On the assessment using the Mini-Mental State Examination,^13^ she had a score of 12/30. In the Digits Span Test, she could repeat 4 digits in direct order, but none in reverse order. She scored 3 on the Semantic Verbal Fluency Test^14^ (animals), while for the Memory of Figures Test,^15,16^remembered 2 items after 5 minutes, but had 3 intrusions from the previous test. She presented with apraxia for both transitive and non-transitive gestures.

**Figure 1:**
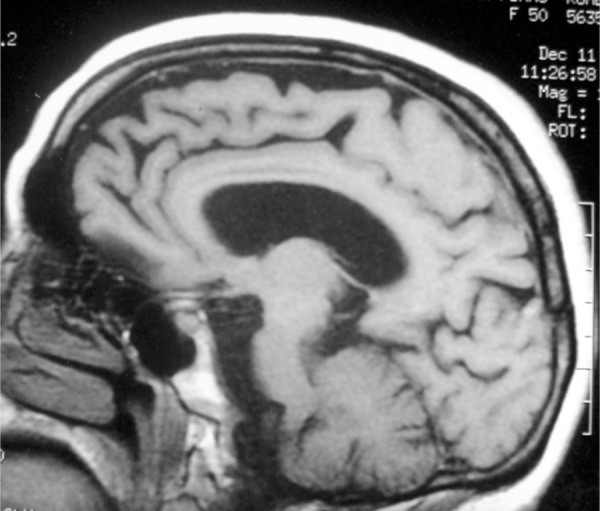
Neuroimage of the patient IPR performed early in the disease.

The Magnetic Resonance Imaging showed global atrophy of the brain with predominance in frontotemporal structures (Figure 1). The SPECT revealed hypoperfusion of frontal, parietal, and temporal lobes, basal ganglia and thalami of moderate / severe degree.

We began phased withdrawal of neuroleptics and introduced an serotonin reuptake inhibitor. The patient developed worsening of aggression without improvement in apathy or motor behavior. As the behavioral disorder was very intense, hampering even her basic care, we began the use of olanzapine.

Ten months after the first assessment, the patient fell from a ladder at her home. She was assisted by her family who lifted her from the ground, after which she showed no pain or any other sequelae of the trauma. On the day following the fall, her husband noticed that her shoulders were asymmetrical with an apparent callousness in the right clavicle region. He took her to the emergency room where she was found to have a complete fracture of the right clavicle. The patient did not accept immobilization, having removed the bandages several times until her family gave up reapplying them.

**Figure 2:**
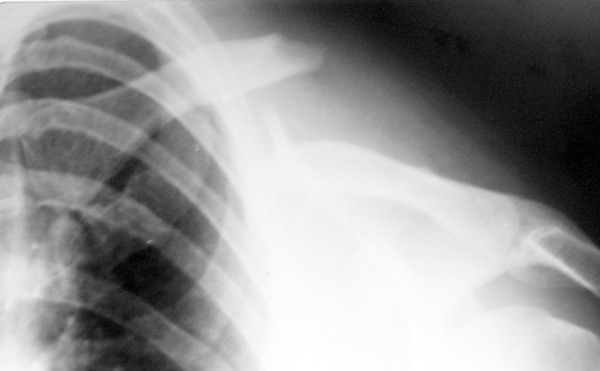
Radiography showing fracture of the clavicle.

At this point she was taken for consultation with our group when we noticed the fracture (Figure 2). The bone fragments were uneven and visible under the skin. During the clinical examination we performed movements of adduction, abduction, flexion and extension of the right upper limb and palpation of the area and we found that the fracture was complete and not yet consolidated. The patient reported pain only when questioned repeatedly, but exhibited no expressions of feeling pain or neurovegetative changes during the examination (change of pupil diameter, increase of the heart and breathing frequency or increase in blood pressure).

The patient did not attempt to evade the painful stimulus, and did not show any change in behavior. She was medicated with analgesics and anti-inflammatory drugs.

At her last visit after two years, the patient still had moderate performance in aspects related to her autobiographical memory. She presented verbal stereotypies, repeating “is it good?” throughout the consultation, interspersed by periods in which she kept her mouth open with extrusion of the tongue. She was unable to sit or lie down without assistance.

The patient was diagnosed with Frontotemporal Lobar Degeneration, given that she initially presented symptoms consistent with diagnosis of the Frontotemporal Dementia and evolved over time with symptoms compatible with Corticobasal Degeneration Syndrome.

## Discussion

Frontotemporal Lobar Degeneration (FTLD) is considered the second most common form of neurodegenerative dementia after Alzheimer’s disease (AD) in presenile individuals.^17-20^

FTLD includes a spectrum of behavioral and cognitive disorders characterized by degeneration of the frontal and anterior temporal lobes.^21^ The Consensus Criteria^22^ for FTLD distinguished three variants of FTLD which reflect the predominant locus of pathology: frontotemporal dementia (FTD), semantic dementia (SD) and progressive non-fluent aphasia (PNFA). The Corticobasal Degeneration and Progressive Supranuclear Palsy are sometimes included in the spectrum of FTLD because they are tau-positive pathologies.^23^

In this case report we focused on the lack of emotional reactivity and evasive motor motivation upon introduction of nociceptive stimuli by this patient with frontotemporal degenerative disease.

Pain is a complex and subjective perceptual experience, incorporating three dimensions: sensory-discriminative, that permits spatial and temporal localization and qualitative/quantitative analysis of the noxious stimulus; affective-motivational, that produces the motivation to terminate, reduce, or evade noxious stimuli; and cognitive-evaluative, that estimates if the sensation is painful, annoying, or pleasing.^24^ Pain has an individual connotation and is influenced by previous experiences.^25^

These aspects of pain experience have distinct underlying supraspinal pathways. Two systems compose phylogenetically different afferent nociceptive pathways: the older pathway runs through the medial region of the brain stem whereas the newer occupies the lateral region. There is little crosstalk between the two systems.^26^

The lateral pain system consists of a spinothalamic tract that projects to the nuclei of the lateral complex of the thalamus and from this structure to primary and secondary somatosensory areas, parietal operculum, and the insula. This system is involved in the sensory-discriminative features of pain.^27^

The medial pain system includes the following tracts: spinothalamic, that projects to the posterior medial and intralaminar thalamic complex; spinoreticular, that projects to the reticular formation; and spinomesencephalic to the periaqueductal grey matter. These systems play an important part in the motivational-affective and cognitive-evaluative features, the memory for pain, and the autonomic-neuroendocrine responses evoked by pain and their function engender motivational inputs to motor and premotor structures so that they may generate behaviors that prevent, reduce or remove pain.^10,27,28^ There are other areas that participate in the medial pain system: other parts of the thalamic complex, the insula, parietal operculum, the secondary somatosensory cortex, the anterior cingulate cortex (ACC), the amygdaloid complex, the hippocampus, and the hypothalamus.^26,27^

The thalamus represents the principal relay structure for sensory information destined for the cortex. It is involved in the reception, integration, and transfer of the nociceptive potential. The ventromedial and posterior nuclei of the thalamus are a part of the medial system and establish connections with the insular and ACC being involved in the affective-cognitive aspects of pain.^26,29^

The ACC is involved in pain processing, selective attention to stimulus in general and is also directly involved in the control of autonomic functions.^30^ Also, it has a reciprocal connection with the amygdala and this may serve to connect a new stimulus with a past emotional experience. It is described as an interface between motor control, motivational drive, and cognition.^1^

Surgical interventions such as cingulotomies and cingulectomies reduce the overall discomfort caused by noxious somatosensory stimuli but do not impair detection of painful sensations^31^ and may produce undesirable cognitive effects, such as impairments to focused attention, intention, and executive function.^32,33^ Therefore, it is reasonable to suppose that lesions of the ACC contribute to reducing the emotional value and motivation to avoid noxious stimuli.^22,34,35^

After a short period of aggressiveness, we noted that apathy was a predominant behavioral symptom of this patient since the onset of the disease.

Snowden et al. (2001),^36^ noted that patients with apathetic type Frontotemporal Dementia may show a loss of response to pain in contrast with patients with semantic dementia that may demonstrate an exaggerated reaction to sensory stimuli.

Zamboni et al. (2008),^37^ evaluated 62 patients with a clinical diagnosis of FTD using voxel-based morphometry of MRI data to explore the association between gray matter loss and severity of apathy or disinhibition in these patients. These authors showed that the severity of the apathy was associated with atrophy in dorsolateral prefrontal, orbitofrontal cortices, ACC and putamen.

Analyzing the lack of affective-motivational component to nociceptive stimulation in this patient would prove complex and most likely fail to specify the neuroanatomic regions affected, largely because this was a case with severe dementia and because the neuroimaging examinations revealed diffuse affection of cortical and subcortical structures. However, we noted that the ACC is an important structure of the medial pain system in that it is responsible for motivational-affective, cognitive-evaluative and autonomic-neuroendocrine features. This structure has connections with prefrontal and premotor cortices which are fundamental for the appropriate choice of motor behavior along with its planning and both time and spatial organization.^34,38^ This is the principal structure involved in the physiopathology of apathy. Thus, we speculate that this case presented a major dysfunction of the ACC which led to the development of apathy and lack of emotional and motivational response to noxious stimuli presented to this patient.
